# An exploratory psychometric network analysis of the college adaptation questionnaire in a sample of first year students

**DOI:** 10.1371/journal.pone.0348816

**Published:** 2026-05-12

**Authors:** Thomas V. Pollet, Gabriele Adomaviciute, Nadine Haggag, Alyson Dodd, Sam G. B. Roberts

**Affiliations:** 1 Department of Psychology, Northumbria University, Newcastle upon Tyne, United Kingdom; 2 School of Psychology, Liverpool John Moores University, Liverpool, United Kingdom; University of Glasgow, UNITED KINGDOM OF GREAT BRITAIN AND NORTHERN IRELAND

## Abstract

The transition to university requires adjustment by students across many aspects of their lives. Multiple instruments have been developed to assess adjustment to life at university. One of the key instruments which has been widely adopted is the College Adaptation Questionnaire (CAQ). This measure is typically applied as a unidimensional measurement instrument. We examined the structure of the CAQ using a sample of 240 first year students at a UK university using psychometric network analyses. In addition, we examined whether the network structure for the items differed between first generation university students and non-first generation students. Using a Clique Percolation method, we found support for four clusters: *satisfaction with university life and support* (e.g., ‘I am very satisfied with the course of my studies’), *social connection* (e.g., ‘Sometimes I feel rather lonely’), *adjustment to university* (e.g., ‘I find it hard to get used to life here’) and *student motivation* (‘Sometimes I want to give it all up’). Network comparison tests suggested that the structure of the psychometric network did not significantly vary according to first generation status. We discuss our findings with reference to capturing student adjustment to university. Our findings suggest that the CAQ should preferably be used as a multidimensional instrument.

## Introduction

Going to university is a major transition which involves changes across multiple life domains [1,2]. New university students need to adjust to a different academic environment with heightened expectations around independent learning, as well as a new social environment with previous social relationships weakening and new ones forming [3–5]. Given the importance of social support in buffering individuals against stressful life events, successfully forming new friendships plays a key role in a successful transition to university (e.g., [6,7]). For example, in students at Canadian universities, the quality of new friendships formed in the first year predicted not just social adjustment to university, but also feelings of attachment to university and academic adjustment [6]. Further, for the 80% of full-time students in the UK who leave their family home to attend university [8], students need to adapt to living independently and managing their own finances [9]. There is a great deal of variation in the degree to which students overcome these challenges to make a successful adjustment to university, which in turn has long-term effects on student wellbeing [10,11] and academic performance and progression [12–15]. Thus, in the UK, the University Mental Health Charter (UMHC) [13] identifies the transition into university and the first year of university as a crucial period for student integration, success, and well-being. As such, UK universities take many steps to try and ensure a successful transition, including a dedicated Induction Week (‘freshers week’) specifically designed to introduce students to the new academic and social environment before the start of the academic term [16].

Successfully adjusting to university influences students’ wellbeing [10,11,13] and academic performance [14,15] in multiple countries [17,18]. Students themselves would like more research on how social and academic adjustment influence mental health [19]. Adjustment to university varies with student characteristics, such as demographic characteristics (e.g., [20]) and neurodiversity (e.g., [21]). One key factor which may affect adjustment to university is being a first-generation student – students whose parents did not attend university. A large body of research demonstrates that compared to students whose parents did attend university, first-generation students feel a lower sense of belonging to university [22,23], are less likely to continue their studies, and have poorer academic performance [24]. Universities have a wide range of university-wide and course-specific activities aimed at ensuring a successful transition to university, both before arrival and throughout the first year of study [13,16,25,26]. An accurate measure of adjustment to university is needed to conduct more research on factors linked to adjustment; identify specific groups of students, such as first-generation students, who may need extra help or support in managing this transition; and evaluate initiatives and interventions designed to provide this support [20,21,27].

Measures of student adjustment to university are typically multidimensional, capturing adjustment in academic, social, personal-emotional and institutional domains [28–31]. In contrast to the 67-item Student Adaptation to College Questionnaire [SACQ; [28]; [29]; [30]] which is lengthy and not freely available to use, the Crombag’s College Adaptation Questionnaire (CAQ, [31]) is a freely available 18-item scale. It has concurrent validity with the widely-used SACQ [32], with significant correlation of *r* = 0.86 between the total scores on the SACQ and the CAQ [33]. The CAQ has been widely used over many decades (e.g., [34–41]). The CAQ’s reliability, based on Cronbach’s α, is typically reported to be good, i.e., .8 or higher (e.g., [35,41–45]). Vlaander found no social desirability issues with this scale and noted that lower adjustment was associated with depression [33,46]. The scale is typically used as a unidimensional construct of overall adjustment to university (e.g., [35,37,39,40,42–45,47–53]). To our knowledge, only a minority of studies have approached the measure as a multidimensional construct [32,46,54]. An early study from 1981 suggested two clusters, one relating to satisfaction and one relating to loneliness [46]. The first cluster, satisfaction, had an α of .77 and the second cluster, loneliness, had an α = .58. The correlation between the two clusters was weak, *r* = .28. A more recent study based on 300 Greek students used exploratory factor analysis to identify a three-factor solution, proposed as Social Adaptation, Attachment, and Emotional-Personal Adaptation to University, but did not report Cronbach’s alphas for these three factors. [32]. Andreou and colleagues used these same three factors in a sample of 89 Greek students with disabilities and reported α of .77 for Social Adaptation, .75 for Attachment and .79 for Emotional-Personal Adaptation to University [54]. Given that the CAQ is most often used as a unidimensional construct and there is only limited information on the psychometric validity of the potential multidimensionality of the CAQ, there is a need for further research on the dimensionality of this scale to inform its suitability to measure student adjustment. Further, other measures, such as the SACQ [30], typically operationalise adaptation to college or university as a multidimensional construct.

Previous studies have used exploratory factor analysis to examine the dimensionality of the CAQ (e.g., [32,46,54]). Here, we use psychometric network analysis [55–58]. We apply this approach rather than Confirmatory Factor Analysis as there is no consensus on the number of expected dimensions and because our work is exploratory in nature, rather than a validation of the CAQ. In this psychometric network analysis approach, the individual items on a scale are the nodes in the network, and the edges (links between the nodes) reflect the conditional associations between the items [56,57]. These conditional associations indicate that the two items are probabilistically dependent, conditional on all other items in the scale, and this is reflected in the weight of the edge between two nodes [56]. Groups of highly connected nodes are then identified, which represent clusters of nodes that have high levels of conditional associations with each other [56,57]. Thus, unlike traditional factor analytic models, which assume correlations among variables are due to unobserved latent factors rather than causal relations between items, psychometric network analysis estimates the observed pairwise relationships between items in a scale, offering insights into their interaction [57–59]. For example, in personality research, rather than assuming the latent variable extraversion causes party-going behaviour or having lots of friends [60], a psychometric approach identifies direct interactions between these items, in that people who go to parties may gain more friends, and people with more friends may get invited to more parties [57,58]. This novel approach has been particularly successful in clinical psychology (e.g., [61–63]) and personality psychology (e.g., [57,64]). Finally, network analysis offers an intuitive and visual representation of the relationships among variables, enabling researchers to better understand and communicate complex patterns [65].

Therefore, the key aim of this study was to use psychometric network analysis to explore the potential dimensionality of the widely-used CAQ [31]. Given that the transition to university is commonly conceptualised as involving adjustment across a number of different domains, including academic, social and institutional [1,2,28], we aimed to examine how the individual items on the CAQ may cluster together to reflect these different domains. As a secondary goal, we examine whether any network structure we find is invariant between first generation students and non-first generation students. Research has found important differences between first-generation and non-first-generation students in both academic performance [24] and sense of belonging [22,23]. Therefore, the transition process may operate differently for these groups of students, with non-first-generation students able to draw on the knowledge of caregivers to let them know what to expect at university, both socially and academically. For first-generation students, a sense of belonging at university on a given day was especially important in predicting attendance and participation in class [22], whereas for non-first-generation students, attendance and participation in class was not related to their daily sense of belonging [22]. Thus, the associations between the psychological and academic transition to university may vary between first-generation and non-first generation students. As such, examining the network structure of the CAQ in first generation and non-first generation students provides a good, exploratory, first test of the invariance of the network structure [56] of the CAQ in different groups of students in whom we may expect the transition process to vary.

## Methods

### Participants

Data were collected between 21-11-2023 and 14-02-2024. The sample size was determined by the available funding to the researchers as well as the time window for the research assistants. 251 students took part, one student withdrew consent after taking part (*n* = 250). Ten participants did not complete the survey, they were excluded from further analysis (*n* = 240, *M* = 18.71; *SD* = 1.33, two did not report their age). The majority of data were collected before Christmas 2023 (81.25%) and is thus from the students’ first semester at university. The majority of students reported that they were British citizens (*n* = 221). The remaining nationalities (*n* = 19) included American (*n* = 4), Indian (*n* = 3), and German (*n* = 2), with other countries only being represented once (e.g., Ghana). In terms of ethnic background, the high-level categories from the 2021 UK census were used [66]. The majority of participants reported being white / white British (*n* = 182) with other participants reporting other ethnic groups as Asian/Asian British (*n* = 22), Black/African/Caribbean/Black British (*n* = 16), Mixed/Multiple ethnic groups (*n* = 9) or other ethnic group (*n* = 11). Of the 240 participants, 82 were men, 149 were women, five were non-binary, and four preferred not to state their gender. When asked about their year of study, 173 stated they were first year students, 75 were foundation year students and 2 stated they were international students. There was a broad representation of programmes studied (Business and Law: *n* = 87; Science, Technology, Engineering (incl. computing), Math: *n* = 55, Psychology / Sports and Exercise Sciences: *n* = 48, Humanities / Social Sciences: *n* = 27, Other (mostly Arts and Design): *n* = 18, Nursing / Social Work and Education: *n* = 5). The majority of students were in halls of residence (54.17%), and over a third reported living with their parent or legal guardian (35.42%).

### Procedure

Two research assistants (GA, NH) approached students on campus at a large University located in the North East of England. They approached either students who were by themselves or those in small groups (approx. five individuals). After providing informed consent and verbally confirming they were a first year student, students completed the survey individually on their own device, a university PC, or a laptop provided by the research assistants. Participants who were recruited in groups were instructed not to communicate with one another during completion. After completion, participants were debriefed and received a £5 Amazon gift voucher. The survey and procedure were approved by the local ethics committee where the study was conducted (ref: 4879).

### Materials

The participants completed an online survey on Qualtrics.com. The participants completed a survey containing the Crombag [31] adaptation questionnaire, a social support network measure, followed by some sociodemographics and questions about the degree studied. The participants also completed further background measures about social network members and themselves, as well as measures on social media use. The full survey is available from the Open Science Framework (OSF). Below, we limit ourselves to the description of the key measures used in this paper. A broader description can be found in [67].

### Crombag (1968)’s Adaptation Questionnaire

This instrument consists of 18 items which were on a 1–7 Likert scale ‌‌(Table 1, ‘Does not apply’ to ‘Applies very much’). It was initially validated with Dutch Students (α = .89). Eight items are positively framed (e.g., *I am very satisfied with the course of my studies*), ten items are negatively framed (e.g., *Sometimes I want to give it all up*). For our study, the instrument had good reliability based on Cronbach’s α = .89. However, reliability as estimated with McDonald’s $\omega_{h}$ was low at .5 [68]. This low value is taken to suggest multidimensionality. It was for this reason and the absence of consensus on the number of dimensions, that we decided to explore a psychometric network analysis approach‌‌.

**Table 1:** Crombag’s Adaptation Questionnaire Items (from Van Rooijen, 1986).

Number	Item
**1**	**I am very satisfied with the course of my studies.**
**2**	**Sometimes I want to give it all up.**
**3**	**I often ask myself what I am doing here.**
**4**	**I would prefer to study somewhere else.**
**5**	**I made many friends here.**
**6**	**I do not feel very at home at the University.**
**7**	**I never feel bored here.**
**8**	**Sometimes I feel discouraged here.**
**9**	**I find life as a student very pleasant.**
**10**	**Sometimes I feel rather lonely.**
**11**	**Sometimes I do not know what to do with my time.**
**12**	**I find it hard to get used to life here.**
**13**	**What I miss here is someone to talk to freely from time to time.**
**14**	**I am very satisfied with my way of life.**
**15**	**If I feel blue, my friends will help me to get out of it.**
**16**	**I find it very difficult to adjust to student life.**
**17**	**I am glad that I came to study here.**
**18**	**I feel very much at home here.**

### First generation student status

As part of the sociodemographic background questions, we asked: *Are you a first generation university student? (By this we mean that neither of your parents (or legal guardians) has had access to university education and completed a degree).* with the following response options: ‘yes’, ‘no’, ‘prefer not to say’, and ‘don’t know’. There was a roughly even split between first-generation students (*n* = 119), and non-first generation students with (*n* = 112). Eight students preferred not to answer this question and one student stated they did not know. For our research question on comparing networks based on first generation students, we excluded these respondents, thus restricting our sample to either first generation or non-first generation students.

### Data analysis

All analyses were conducted in R 4.5.2 [69]. The key packages used are bootnet [59], qgraph [70], NetworkComparisonTest [71], and CliquePercolation [72]. The data, code, pre-registered analysis plan, and analysis document are available from the Open Science Framework (OSF). The analysis document also contains supplementary analyses, including bootstrapping [73], reports for node measures such as centrality, and alternative methods of community detection (e.g., Louvain method [74]). Given the exploratory focus, we focussed on psychometric network methods, rather than confirmatory factor analysis. These methods allow for intuitively modelling and visualising complex interactions between items. In addition, work by Golino and Epskamp [75] suggested that community detection methods partial correlation network methods may outperform other methods for the discovery of latent dimensions.

The network was estimated with the Graphical Lasso (GLasso) algorithm, with the Extended Bayesian Information Criterion (EBIC) was used for model selection, with the default settings (tuning parameter of .5). The default tuning parameter is suggested to be a good choice [70]. This model is routinely applied (e.g., [76,77]) and at a low sample size where we want to identify the strongest edges a regularised method, such as EBICglasso, is preferred (e.g., [59,78]). The package bootnet is able to accommodate a range of data structures via cor_auto. In our case, the data were identified as ordinal and modelled as such. The models were run in duplicate with different starting seeds and showed convergent results. Items were not reverse scored as this is unnecessary and just implies a different sign.

Our focus is on the grouping of items in this psychometric network rather than the existence of specific edges. The estimation method we used, EBICGlasso, generally performs well for this problem [79]. In order to determine grouping (‘communities’) we used the CliquePercolation method [72], which, unlike other methods (e.g., Louvain [74]), allows nodes to be unassigned or to be assigned to more than one cluster. This method has been successfully applied to identify clusters in similar psychometric studies (e.g., [76,80,81]). We used lavaan [82] and ran a structural equation model (WLSMV estimation) to obtain the latent correlations between the clusters obtained via the CliquePercolation method. Finally, we evaluated whether the networks were invariant according to first generation student status. For this network comparison test, we performed 10,000 permutations.

## Results

**Fig 1:**
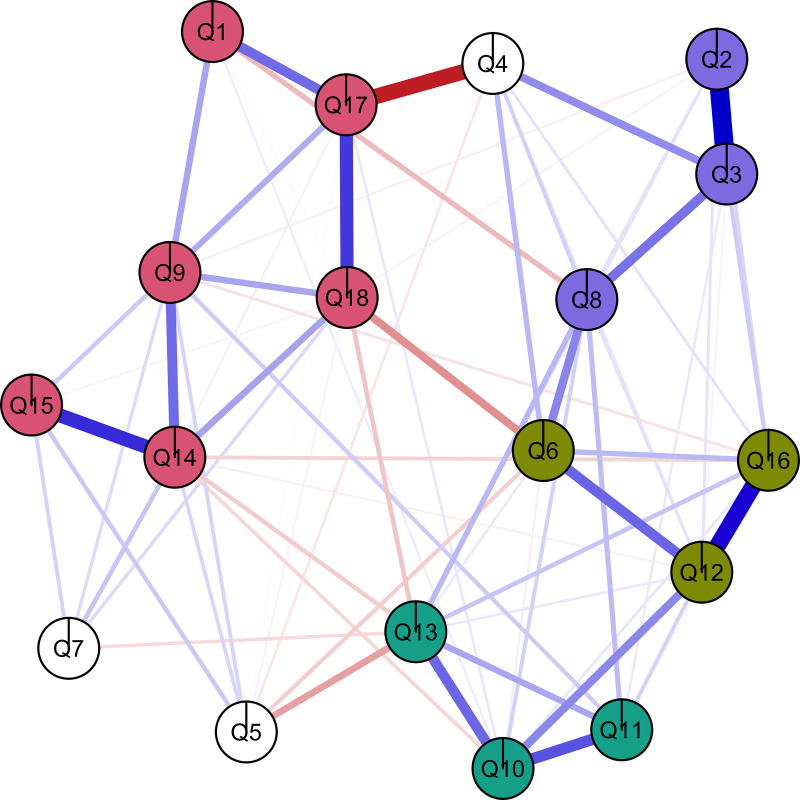
Crombag Questionnaire communities based on Clique Percolation.

The algorithm extracted a network with 76 edges. A common heuristic is three observations per parameter, and our analysis exceeded this heuristic (76 edges * 3 = 228, *n* = 240) [59,83]. Fig 1 shows the spatial layout, and we can observe the strength of the associations between items via the thickness of the lines. Some associations stand out. For example, Q2: *Sometimes I want to give it all up.* and Q3: *I often ask myself what I am doing here*, Q14: *I am very satisfied with my way of life.* and Q15: *If I feel blue, my friends will help me to get out of it.* and Q12: *I find it hard to get used to life here.* and Q16: *I find it very difficult to adjust to student life.*. There is also a strong negative relationship between Q4: *I would prefer to study somewhere else* and Q17: *I am glad that I came to study here*

The CliquePercolation procedure led to the identification of four clusters (Fig 1). In Fig 1, positive associations are represented in blue and negative associations in red, and the size of the edge is proportional to the strength of the association. Three items were unassigned to one of the clusters (item 4: I would prefer to study somewhere else, item 5: I made many friends here, item 7: I never feel bored here, blank nodes in Fig 1). Table 2 contains the items and the proposed grouping: one cluster with six items, the remaining three clusters with three items each. We tentatively label the first cluster: satisfaction and support (*Pink* in Fig 1). The second cluster captures items relating to social connection, and we therefore use this label (*Teal* in Fig 1). The third cluster refers more broadly to adjustment to university life (*Camo green* in Fig 1). The fourth cluster refers largely to items referring to student motivation (*Purple* in Fig 1). The clusters are relatively separate, though the negative association between Q18: *I feel very much at home here.* and Q6: *I do not feel very at home at the University.* suggests that these items potentially form a bridge between clusters.

**Table 2:** Crombag’s Adaptation Questionnaire Items: Clustering.

Number	Item
**Satisfaction and support**	
**1**	**I am very satisfied with the course of my studies.**
**9**	**I find life as a student very pleasant.**
**14**	**I am very satisfied with my way of life.**
**15**	**If I feel blue, my friends will help me to get out of it.**
**17**	**I am glad that I came to study here.**
**18**	**I feel very much at home here.**
**Social connection**	
**10**	**Sometimes I feel rather lonely.**
**11**	**Sometimes I do not know what to do with my time.**
**13**	**What I miss here is someone to talk to freely from time to time.**
**Adjustment to university life**	
**6**	**I do not feel very at home at the University.**
**12**	**I find it hard to get used to life here.**
**16**	**I find it very difficult to adjust to student life.**
**Student motivation**	
**2**	**Sometimes I want to give it all up.**
**3**	**I often ask myself what I am doing here.**
**8**	**Sometimes I feel discouraged here.**
**Unassigned**	
**4**	**I would prefer to study somewhere else.**
**5**	**I made many friends here.**
**7**	**I never feel bored here.**

Table 3 contains the latent correlations between the four proposed clusters. Two correlations exceeded |.70|: Social Connection and Adjustment to University Life (1) and Student Motivation and Adjustment to University Life (2). However, the other correlations were below |.70|, suggesting the clusters may represent distinct aspects of the adjustment to university.

**Table 3:** Latent factor correlations.

	Latent Factors			
	Satisfaction and Support	Social Connection	Adjustment to University Life	Student Motivation
**Satisfaction and Support**	1.000			
**Social Connection**	−0.412	1.000		
**Adjustment to University Life**	−0.612	0.755	1.000	
**Student Motivation**	−0.451	0.641	0.710	1.000

Model estimated via WLSMV estimator in lavaan.

### Network comparison: First generation status

The CliquePercolation algorithm yielded four clusters for both groups, as in the pooled data (Table 2). The network was invariant between the first generation student group and non-first generation group (*M* = 0.249, *p* = .631). This suggests the layout between the first generation students and the non-first generation status are broadly similar. The global strength invariance (*S* = 1.508, *p* = .053) test was not statistically significant at *p* = .05. The strength was somewhat greater for the first generation students (7.92) versus non-first generation students (6.41). Phrased differently, albeit not significantly, this suggests that the network connectivity between the items is greater for the former than for the latter.

## Discussion

The aim of this study was to use psychometric network analysis to examine the structure of the College Adaptation Questionnaire (CAQ) [31] in a sample of UK first year university students. There were two key findings. First, the network analysis identified four clusters, which represent groups of items which correlate highly together. These clusters were fairly independent (Table 3). The largest cluster of six items related to *satisfaction with university life and support* (e.g., ‘I am very satisfied with the course of my studies’). There were three smaller clusters of three items each related to *social connection* (e.g., ‘Sometimes I feel rather lonely’), *adjustment to university* (e.g., ‘I find it hard to get used to life here’) and *student motivation* (‘Sometimes I want to give it all up’). There were three items which were not allocated to any cluster (*Q4*: *‘I would prefer to study somewhere else.’*, *Q5*: *‘I made many friends here.’*, *Q7*: *‘I never feel bored here’.*). These three items were toward the periphery of the network. One of these items, ‘I would prefer to study somewhere else’, was very strongly inversely related to ‘I am glad that I came to study here’ (Q17). This suggests redundancy. Though further work is needed, if researchers face space/time limitations, they could consider not including these three unassigned items. Second, this psychometric network structure was very similar for the first generation (*n* = 119) and non-first generation students (*n* = 112). This suggests the correlations between the items in the four clusters are similar for these two groups of students, despite the different challenges these students face in their transition to university [22–24].

These results have important implications for future studies as they challenge the unidimensionality of the CAQ. The CAQ is a widely-used measure of how students adjust to university life (e.g., [34–41]) and is typically used as a unidimensional scale (e.g., [35,37,39,40,42–45,47–53]). If used as a unidimensional measure, it would seem that the measure largely captures ‘satisfaction and support’ as this is the largest cluster. This might have consequences for what findings on the CAQ mean. Further, the unidimensionality of the CAQ is often assessed with Cronbach’s alpha. This does not actually provide evidence of the unidimensionality of a scale, but instead *assumes* unidimensionality in its calculation [84]. Therefore, although previous studies have found ‘good’ reliability of the scale with Cronbach’s alphas of over 0.8 (e.g., [35,41–45]) and the alpha for this study was .89, these reliability estimates do not in themselves demonstrate that the scale has a unidimensional structure [84]. In contrast, McDonald’s $\omega_{h}$ is a more general estimate of scale reliability than Cronbach’s alpha, as it does not assume ‘tau equivalence’ – that all items on a scale contribute equally to the overall construct [84]. For this study, the $\omega_{h}$ for CAQ was low at 0.5 [68], suggesting multidimensionality. However, as many studies only report α it is difficult to gauge how widespread this issue is.. In sum, in spite of a strong overall α, the measure might still be multidimensional. With some exceptions, the latent correlations between the dimensions we found suggest these dimensions are to be treated as distinct. The network analysis also allows for some additional insights. The graph shows that central items are Q18 (‘I feel very much at home here’) and Q6 (‘I do not feel very at home at the University.’). This suggests that in cases where researchers are severely limited and can only rely on a single item measure, these two items potentially make good candidates. These also align with the concepts of social connection and belonging, which are linked to academic and well-being outcomes [85].

In line with the University Mental Health Charter [13], the suspected multidimensionality of the CAQ points toward the importance of different facets of adjusting to university: from academic, to social, to the transition itself. Although the CAQ is relatively old, the clusters correspond to contemporary research on factors influencing student outcomes and the multifaceted nature of the transition to university [2,4,86]. Whilst the four factors were clearly distinguished in the analysis, there were also links between them (see Fig 1) and research suggests they are likely to be inter-related. For example, forming and maintaining new friendships at university is associated with both adjustment to university life [6,7] and academic performance [87]. In our model, the ‘adjustment to university life’ cluster is linked to all three of the other clusters (satisfaction and support, social connection and student motivation), and this was consistent across first-generation and non-first-generation students. Further, one advantage of psychometric analysis over factor analysis is the estimates of the conditional associations between pairs of items [58]. For example, in our model (Fig 1), there was a strong pairwise association between Q14 (‘I am very satisfied with my way of life’) and Q15 (‘If I feel blue, my friends will help me get out of it’) as indicated by the thickness of the line between these two nodes. From a network perspective, the interpretation is that these items may directly influence each other, rather than assuming an unobserved latent dimension of ‘satisfaction and support’ is responsible for the strong association between these items, as in a factor analysis approach. In university students, there is an association between strong social support and life satisfaction [88,89]. Given the basic and universal human need to form stable, interpersonal relationships with others – a ‘need to belong’ [90] – a sense of being supported by friends (Q14) may directly influence life satisfaction (Q15), rather than operating through a shared latent variable of ‘satisfaction and support’.

Overall, therefore, our study suggests the CAQ has a multidimensional rather than unidimensional structure, with links across the different clusters and with individual items. As is the case for well-being [86], reliable measures of constructs related to adjustment to university life are necessary to investigate the impact of student transitions and evaluate initiatives designed to support this journey. Evidence for the importance of this multidimensional approach comes from a meta-analysis (*k* = 237, *N* = 44,668) of how the different factors of the SACQ relate to academic performance and students’ traits [12]. For example, Grade Point Average (GPA) was most strongly related to academic adjustment as compared to personal-emotional adjustment, whilst depression was strongly related to personal-emotional adjustment as compared to academic adjustment. Further, for the CAQ specifically, in a study treating this scale as a multi-dimensional construct, the personal-emotional adaptation subscale was significantly associated with depression, whereas the social adaptation subscale was significantly associated with life satisfaction [54]. Thus, the different dimensions of adjustment to university are associated with distinct student outcomes, suggesting it is important to accurately measure the multidimensional nature of adjustment to university.

Clearer operationalisation of adjustment can also aid with conceptualisation, helping staff to understand the different domains of adjustment and what students require support with to navigate the transition into and through university. For example, most academic staff are Personal/Guidance Tutors, and students perceive them as vital support during transitions [91]. Understanding different areas of adjustment (e.g., adjustment to the university as a whole vs. social connection) could form points of discussion in Personal/Guidance Tutor meetings and help staff to signpost students to further support where needed, in recognition that staff lack confidence and clarity in how to use these meetings [92]. This is also important because if support and interventions are developed aiming to enhance student adjustment, measures relying solely on a unidimensional construct could miss more nuanced outcomes. For example, of the four clusters of the CAQ, a campaign targeting loneliness could make a meaningful impact on social connection, but might have a limited impact on student motivation. Refining our measures and making more specific predictions for interventions could thus be beneficial to evaluate real, measurable change.

It is important that we can measure different areas of adjustment, as some students might be doing well in some but not others, and an overall score might not pick up if they are struggling and what they are struggling with. Research on determinants of adjustment, as well as initiatives to support students during transitions, can focus on these different domains to identify more specific relationships. This can include work on social determinants of adjustment, where some students from more disadvantaged backgrounds might struggle with adjustment in specific areas [10,21,93]. This means we can design and evaluate more nuanced interventions and signpost them to students who need them most. For example, in the context of student counselling, being able to differentiate between student issues relating to motivation versus social connectedness could influence the support offered. Likewise, a good multidimensional measure might be helpful to programme leaders. If such a measure identifies primarily issues with social connection rather than, say, motivation, then different changes might be sought. For example, developing more interactive course work in smaller groups in the context of low social connection, as opposed to trying to implement different motivational strategies (e.g., increasing positive feedback) in the context of low motivation.

As well as being an important student outcome, adjustment is also a predictor of further outcomes such as student engagement and well-being [15]. In fact, these are likely to be complex, bidirectional associations. Again, understanding and being able to measure the multidimensional nature of student adjustment is important for disentangling more specific components impacting different areas of student life.

### Limitations

The sample size, though suitable for network analysis, is likely not large enough to conduct further psychometric testing with, for example, techniques from item response theory [94] or other network estimation methods. Further, generalisability is limited due to all participants being recruited from a single university in the UK. Whether the structure we uncovered for the CAQ generalises to students from other universities, and also those in other countries, remains to be tested. We call for research with more generalisable samples. The best design would cover a range of universities with representative samples from each university. Finally, this study was not designed to be a full-blown psychometric evaluation of the CAQ. Instead, it is an exploratory secondary data analysis motivated by our finding that the CAQ did not conform to a unidimensional structure.

### Future directions

Our analyses were exploratory, relying on exploratory graph analyses via the clique percolation method. Confirmatory analyses are necessary to validate the proposed structures. In addition, further psychometric testing in a larger, more representative sample could include evaluating other aspects of invariance, apart from first-generation status. Other avenues for assessing invariance could be comparing different study subjects (e.g., STEM vs. Non-STEM) or other student characteristics (e.g., Home versus international students; part-time versus full-time students). More work is also needed on the temporal stability of the CAQ and its clusters. Some clusters might be more temporally stable than others – for example, the social connection cluster could be susceptible to change based on the time of the year (e.g., before or after the Christmas break). The majority of our data were collected before the Christmas break (>80%) and thus does not allow for a closer inspection of how the CAQ changes over time. Finally, it would be worthwhile examining whether, in a real-world context, treating the CAQ as unidimensional versus multidimensional leads to fundamentally different conclusions when interventions aiming to improve adjustment are implemented.

## Conclusion

While still widely used, the CAQ was developed in the late 1960s. Nonetheless, the existing items map on to areas in up-to-date research as well as priority areas of the University Mental Health Charter [13]. Thus, they are still relevant, and the use of an existing measure and identifying key components within that measure is good for parsimony and comparison across research. However, there may be further areas of adjustment relating to current student demands and concerns. Student co-production could be used to identify key domains of adjusting to university to expand on the existing measure. For now, we suggest that the CAQ remains a key candidate measure for use in studies exploring the student experience at university. However, our findings suggest that it should be preferably conceptualised as a multidimensional measure, rather than as a unidimensional one.
